# Folate Receptor Expression by Human Monocyte–Derived Macrophage Subtypes and Effects of Corticosteroids

**DOI:** 10.1177/19476035221081469

**Published:** 2022-03-07

**Authors:** Kelly Warmink, Michiel Siebelt, Philip S. Low, Frank M. Riemers, Bingbing Wang, Saskia G. M. Plomp, Marianna A. Tryfonidou, P. René van Weeren, Harrie Weinans, Nicoline M. Korthagen

**Affiliations:** 1 Department of Orthopedics, University Medical Center Utrecht, Utrecht, The Netherlands; 2 Department of Orthopedics, Erasmus Medical Center, Rotterdam, The Netherlands; 3 Department of Chemistry, Purdue University, West Lafayette, IN, USA; 4 Department of Equine Sciences, Faculty of Veterinary Medicine, Utrecht University, Utrecht, The Netherlands; 5 Department of Biomechanical Engineering, TU Delft, Delft, The Netherlands

**Keywords:** corticosteroids, disease marker, FR-beta, M1/M2, macrophages

## Abstract

**Objective:**

Folate receptor beta (FR-β) has been used as a clinical marker and target in multiple inflammatory diseases, including osteoarthritis (OA) and rheumatoid arthritis (RA). However, the conditions under which FR-β^+^ macrophages arise remain unclear and could be affected by corticosteroids. Therefore, we studied FR-β expression *in vitro* in macrophage subtypes and determined their response to triamcinolone acetonide (TA), a clinically often-used corticosteroid.

**Design:**

Human monocyte–derived macrophages were differentiated to the known M0, M1, or M2 macrophage phenotypes. The phenotype and FR-β expression and plasticity of the macrophage subtypes were determined using flow cytometry, reverse transcription-quantitative polymerase chain reaction (RT-qPCR), and enzyme-linked immunosorbent assay (ELISA).

**Results:**

FR-β expression was low in granulocyte-macrophage colony-stimulating factor (GM-CSF)-generated (M1-like) macrophages and high in macrophage colony-stimulating factor (M-CSF)-generated (M0 and M2-like) macrophages. FR-β expression remained high once the M0 or M2 macrophages were stimulated with pro-inflammatory stimuli (interferon-γ plus lipopolysaccharide) to induce M1-like macrophages. On the contrary, anti-inflammatory TA treatment skewed GM-CSF macrophage differentiation toward an M2 and FR-β^+^ phenotype.

**Conclusions:**

As corticosteroids skewed monocytes toward an FR-β-expressing, anti-inflammatory phenotype, even in an M1 priming GM-CSF environment, FR-β has potential as a biomarker to monitor success of treatment with corticosteroids. Without corticosteroid treatment, M-CSF alone induces high FR-β expression which remains high under pro-inflammatory conditions. This explains why pro-inflammatory FR-β^+^ macrophages (exposed to M-CSF) are observed in arthritis patients and correlate with disease severity.

## Introduction

Macrophages are heterogeneous immune cells that play an important role in tissue homeostasis and innate immunity. Most macrophages develop from peripheral blood monocytes that have migrated to local tissues, although tissue-resident macrophages have also been identified.^1,2^ Macrophages are implicated in both the pathology and resolution of a wide range of diseases, including cancer, atherosclerosis, autoimmune disorders, and osteoarthritis (OA) and rheumatoid arthritis (RA).^3^ Within the field of (osteo)arthritis, macrophages play an important role in inflammatory processes in the joints.^4,5^ Their role is strongly dependent on their phenotype. Distinct macrophage phenotypes have been identified *in vitro* based on their surface markers, each with their own function in inflammation, wound healing, and immune regulation.^6,7^ Classically activated pro-inflammatory macrophages are known as M1. They can produce many pro-inflammatory cytokines including tumor necrosis factor (TNF)-α, interleukin (IL)-6, and IL-12 and are hallmarked by the expression of surface marker CD80. Alternatively activated anti-inflammatory macrophages are known as M2 and are generally identified by expression of CD163, arginase 1, and/or CD206, depending on their subtype. Macrophages can be generated *in vitro* from monocytes by stimulation with granulocyte-macrophage colony-stimulating factor (GM-CSF) or macrophage colony-stimulating factor (M-CSF), which are often used to prime toward M1 or M2 phenotypes, respectively. Although the M-CSF-generated macrophage primarily differentiates into an M2-like phenotypic and functional properties, they remain very plastic and can be skewed to an M1-like phenotype by stimulation with interferon (IFN)-γ and lipopolysaccharide (LPS). Therefore, the M-CSF-generated macrophage is often referred to as M0 phenotype. Further subtypes for M2 macrophages have been described that are induced *in vitro* by stimulation of M0 macrophages, such as M2a (tissue repair) macrophages that are induced by stimulation with IL-4, IL-10, and IL-13. M2b (regulatory) macrophages are induced by immune complexes (ICs) in combination with LPS, which results in the production of IL-10 and IL-6. Finally, the M2c (immunosuppressive) subtype is induced by corticosteroids, IL-10, and transforming growth factor (TGF)-β.^7,8^ However, it is becoming clear that *in vivo* macrophages are not dichotomously classifiable in terms of M1 and M2, but are more plastic, and can even simultaneously express M1 and M2 markers.^7,9^ In addition, therapeutic agents can affect macrophages directly and alter their phenotype. Corticosteroids, which are commonly used in inflammatory joint diseases, can drive macrophages toward an M2 phenotype based on upregulated CD163 surface expression, production of anti-inflammatory cytokine TGF-β, and increased IL-10 gene expression.^10-12^

Macrophages have been found to specifically express folate receptor beta (FR-β), a member of a family of receptors with high affinity for physiological folates. Because of this, folate receptor–based imaging and therapeutic approaches (often originally developed for tumors expressing FR-α) have been evaluated for their use in macrophage-mediated diseases.^13-17^ FR-α/β-specific tracers have been developed for gamma scintigraphy and SPECT (EC20 FolateScan),^18-21^ PET,^22,23^ and fluorescence imaging,^24^ and are being assessed for their possible diagnostic use in RA and OA.^20,25^ FR-based agents are already widely used to monitor macrophage activation in animal models for RA and OA.^21,26,27^ The specific surface expression of FR-β on macrophages also provides a potential target in these diseases for the use of FR-linked drugs or FR-β-directed immunotherapy.^28-31^

The clinical implementation of FR-targeted imaging and therapies is, however, hampered by the fact that it remains unclear which macrophage phenotypes express FR-β. Furthermore, accurate monitoring of disease activity and therapeutic responses in patients requires knowledge about the possible interactions with clinically commonly applied drugs, such as corticosteroids. Interaction between corticosteroids and FR-β expression may have significant implications for the use of FR-based agents in (osteo)arthritis or other inflammatory and autoimmune diseases, in which corticosteroid use is part of standard of care. Therefore, this study aimed to identify FR-β expression in currently recognized macrophage subtypes and to assess the effects thereon of triamcinolone acetonide (TA), a clinically commonly used corticosteroid.

## Methods

### *In Vitro* Differentiation of Monocyte-Derived Macrophages

Blood from healthy human donors (*n* = 15) was supplied by the Dutch blood bank (Sanquin, Amsterdam) in accordance with applying laws and ethical guidelines and under legal agreement (number 150731). Peripheral blood mononuclear cells (PBMCs) were isolated from peripheral blood using Ficoll-Paque (Pharmacia, Uppsala) density centrifugation. Monocytes were positively selected by magnetic-activated cell sorting (MACS) with anti-CD14-labeled microbeads (Miltenyi Biotec, Utrecht) according to manufacturers’ instructions using the autoMACS (Miltenyi Biotec). Purity of the monocytes was evaluated on flow cytometry by assessing the percentage of CD14+-stained cells, which was on average 92.4% ± 4.5%.

Monocytes were cultured in RPMI/glutamax (Gibco BRL, Life Technologies) with additional pen/strep (100 U/ml penicillin and 100 μg/ml streptomycin; Gibco), and 10% v/v fetal calf serum (FCS; Gibco BRL) at a cell density of 500,000/well in 24-well plates (Nunclon Delta; Thermo Scientific, Waltham, MA). Monocyte-derived macrophages were generated by culturing monocytes for 7 days in the presence of 800 U/ml human recombinant GM-CSF (M1 priming cytokine) or 25 ng/ml human recombinant M-CSF (M2 priming cytokine, both from R&D Systems, Minneapolis, MN).^11,32^ Subsequently, macrophages were stimulated for an additional 24 hours to create the desired macrophage phenotype. For M1 macrophages, GM-CSF-generated macrophages were stimulated with 10 ng/ml IFN-γ and 10 ng/ml LPS, further referred to as M1(IFN+LPS). For the M2 macrophage subtypes, M-CSF-generated macrophages were stimulated with 10 ng/ml IL-4 and 10 ng/ml IL-10 (M2a), 50 μg/ml ICs and 10 ng/ml LPS (M2b), or 10 ng/ml TGF-β (M2c), further referred to as and M2a(IL4+IL10), M2b(LPS+ICs), and M2c(TGF-β), respectively.^11,32-34^ IFN-γ, IL-4, IL-10, and TGF-β were all from PeproTech (Rocky Hill, NJ, USA), LPS from Sigma-Aldrich (Saint Louis, MO, USA). M-CSF- and GM-CSF-generated macrophages where no stimulation was added during the additional 24 hours of culture are referred to as M-CSF control and GM-CSF control. ICs were produced according to the method of Ottonello *et al.*^34^ by incubating human albumin (15 μg/ml) with rabbit anti-human albumin (150 μg/ml) at 37 °C for 1 hour and then overnight at 4 °C. The precipitated ICs were washed twice in phosphate-buffered saline by centrifugation (5,000*g*, 10 minutes) and then stored at 4 °C.

The effect of an inflammatory stimulus on M-CSF-generated macrophages was studied by stimulating with 10 ng/ml IFN-γ and 10 ng/ml LPS or 10 ng/ml LPS only for 24 hours. To study the effect of the anti-inflammatory drug TA, 100 nM TA (Kenacort; Bristol-Myers Squibb, Woerden) was added to the medium on day 0, day 3, or day 7 in the cell culture with M-CSF or GM-CSF, no additional stimulators were added at day 7. On day 8, supernatants and cells were harvested for further analysis. The culture medium was refreshed after 3 to 4 days. Macrophage subtypes were analyzed with the aid of surface markers via flow cytometry, gene expression analysis, and cytokine measurement on protein level.

### Flow Cytometry

Membrane markers to characterize the macrophage subtypes and FR-β were analyzed using immunofluorescent flow cytometry by incubating the cells with specific fluorescent antibodies diluted in phosphate-buffered saline + 1% w/v bovine serum albumin + 0.1% v/v sodium azide + 1% v/v normal rabbit serum (all from Sigma-Aldrich). Cells were detached from the culture plate by scraping, no trypsin was used. Macrophages were first incubated at 4 °C for 30 minutes with biotinylated M909, a biotin-labeled human monoclonal antibody that specifically binds human FR-β,^29^ followed by incubation with a fluorescein (FITC)-labeled streptavidin (BD Biosciences, San Jose, CA), CD80-PE (Clone L307.4; BD Biosciences), CD163-PerCP-Cy5.5 (clone GHI/61; Biolegend, San Diego, CA), CD14 APC-AF750 (clone RMO52; Beckman Coulter, Brea, CA), CD206-PC-7 (clone 3.29B1.10; Beckman Coulter), and CD16-APC (clone 3G8; Life Technologies, Frederick, MD). Alternatively, CD68-FITC (EBM11; DAKO, Denmark) or CD86-PE (clone IT2.2; BD Biosciences) was included. Dead cells were excluded using viability marker SYTOX (ThermoFisher, Waltham, MA). More details on the antibodies and gating strategy that were used are shown in Suppl. Table S1 and Suppl. Figure S1. Flow cytometry was performed on a FACSCanto II cytometer (Becton Dickinson) according to the manufacturer’s protocols. Fluorescence minus one controls were used to confirm there were no false-positive populations. Values were expressed as mean fluorescence intensity (MFI); at least 6 donors were used for all flow cytometry experiments.

### Detection of messenger RNA Levels by reverse transcription-quantitative polymerase chain reaction

Messenger RNA (mRNA) was isolated using RNeasy Mini Kit (Qiagen, Venlo). After on-column DNase-I treatment (Qiagen RNAse-free DNase kit), RNA was quantified using Nanodrop ND-1000 (Isogen Life Science, De Meern) and reverse transcribed into cDNA using the iScript cDNA Synthesis Kit (Biorad, Veenendaal). Gene expression was determined using the CFX384 Real-Time PCR Detection System (Biorad). The quantitative polymerase chain reaction (qPCR) reactions were performed in duplicate in 384-well plates in a final volume of 10 μl, using IQ SYBR Green Supermix (Biorad). The mRNA levels were normalized to those of the reference genes *TBP* and *YWHAZ* using the delta Ct method. At least 6 donors were used in all qPCR measurements. Details on the primers that were used for reverse transcription-quantitative polymerase chain reaction (RT-qPCR) are shown in Suppl. Table S2.

### Cytokine Measurements

Cytokine production was measured in supernatants after 24 hours stimulation. TNF-α, IL-6, IL-10, and IL-12 (p40/p70) were measured using novex cytoset enzyme-linked immunosorbent assay (ELISA) kits (ThermoFisher) according to the manufacturer’s recommendations. Analysis was performed using an iMARK microplate absorbance reader and Microplate Manager 6 software (Bio-Rad). At least 5 donors were used in all cytokine measurements.

### Statistics

Data were analyzed using GraphPad Prism version 7.04 (La Jolla, CA) and SPSS Statistics for Windows, version 25.0 (IBM Corp., Armonk, NY). A generalized linear mixed model was used to compare differences between groups; depending on the distribution of the data, either a normal distribution with identity link or a gamma distribution with log link was used. The Bonferroni method was used to correct for multiple comparisons and *P* values <0.05 were considered statistically significant. Confidence intervals (CI) are given at the 95% level. Results are shown as mean ± standard deviation.

## Results

### Marker Expression in Macrophage Subtypes

**Figure 1.**
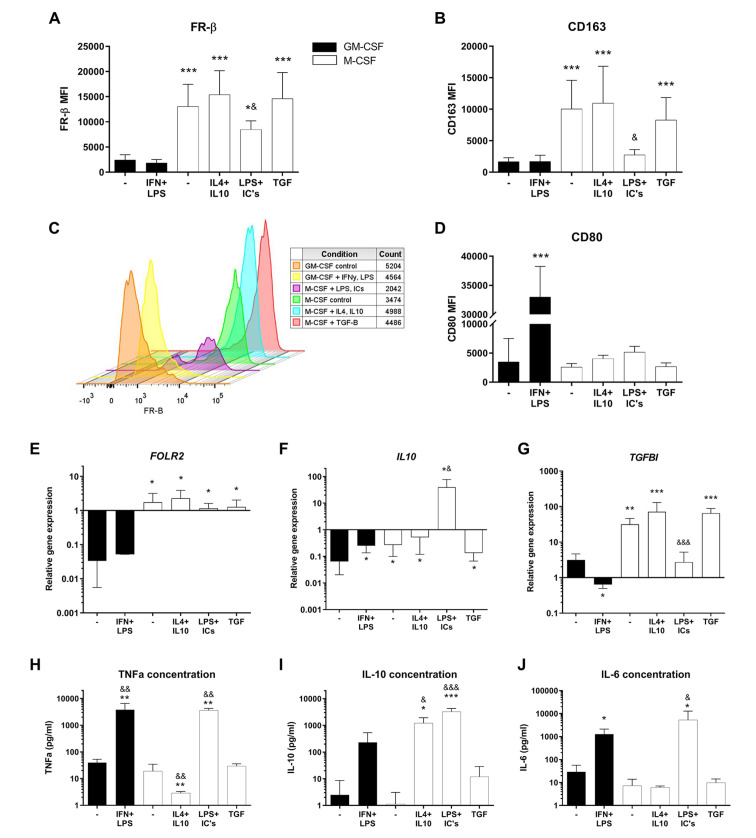
FR-β surface expression and other characteristics of macrophage subtypes. Experimental conditions consist of GM-CSF (black bars) or M-CSF (white bars) generated macrophages, stimulated for 24 hours with corresponding cytokine(s). (**A-D**) Mean fluorescence intensity (MFI) of FR-β, CD163, and CD80 as measured by flow cytometry. (**E-G**) Relative gene expression of FOLR2, IL10, and TGFBI, calculated using delta Ct method and housekeeping genes TBP and YWHAZ. (**H-J**) Cytokine concentration of TNF-α, IL-10, and IL-6 in culture supernatants measured by ELISA. Graphs represent mean ± SD. *P < 0.05, **P < 0.01, ***P < 0.001 compared with GM-CSF control; ^&^*P* < 0.05, ^&&^*P* < 0.01, ^&&&^*P* < 0.001 compared with M-CSF control, as determined by generalized linear mixed models with Bonferroni correction. FR-β = folate receptor beta; GM-CSF = granulocyte-macrophage colony-stimulating factor; M-CSF = macrophage colony-stimulating factor; TGFBI = transforming growth factor beta induced; TNF = tumor necrosis factor; IL = interleukin; ELISA = enzyme-linked immunosorbent assay.

FR-β surface expression was markedly higher in all M-CSF (M2-like) generated macrophage subtypes compared with GM-CSF (M1-like) generated macrophages (**Fig. 1A** and **C**; *P* < 0.05 for all groups). Although the M2b(LPS+ICs) macrophages showed lower FR-β surface expression compared with the other M-CSF groups (*P* < 0.05 for all 3 groups), it was still significantly higher than in GM-CSF-generated macrophages (*P* < 0.05). Phenotypes of the macrophage subtypes were confirmed with the aid of surface marker flow cytometry analysis. M-CSF-generated macrophages displayed higher CD163 expression compared with GM-CSF-generated macrophages (**Fig. 1B**; *P* = 1.8 × 10^−6^, CI = 4,022-12,709) and here too CD163 expression by the M-CSF-generated macrophages was lowest upon stimulation with LPS and ICs to induce an M2b subtype (*P* < 0.05 compared with M-CSF control). These results indicate that, in M1 and M2 *in vitro* macrophage subtypes, the FR-β receptor expression pattern is similar to the expression of traditional M2-like marker CD163. GM-CSF-generated macrophages displayed higher surface expression of CD80 after stimulation with IFN-γ and LPS compared with GM-CSF control (*P* = 1.0 × 10^−15^, CI = 23,612-35,259) and compared with all M-CSF-generated subtypes (**Fig. 1D**; *P* < 0.001 for all M-CSF groups). When looking at the CD14 and CD16 surface expression, only CD16 showed an increase in expression in the M2a(IL4+IL10) group (*P* = 0.0002) and a decrease in expression in the M2b(LPS+ICs) group (Suppl. Fig. 2C; *P* = 0.01).

In accordance with the FR-β surface marker, the FR-β (*FOLR2*) relative gene expression was also significantly higher in M-CSF compared with GM-CSF-generated macrophages (**Fig. 1E**; *P* < 0.05 for all M-CSF subtypes), with no difference between the M-CSF subtypes. To confirm the M2, anti-inflammatory, phenotype in M-CSF-generated macrophages, *IL10* and TGF-β-Induced Protein (*TGFBI*) gene expression were quantified. The *IL10* gene expression was significantly increased in all M-CSF-generated macrophages compared with GM-CSF control (**Fig. 1F**; *P* < 0.05) and particularly high in M2b(LPS+ICs) macrophages. The same was true for the *TGFBI* expression, with the exception of the M2b(LPS+ICs) macrophages, where the expression level was comparable with that of the GM-CSF control and significantly lower than that of the other M-CSF macrophage subtypes (**Fig. 1G**; *P* < 0.001 for all M-CSF subtypes).

To unequivocally confirm the phenotype of the M2b(LPS+ICs) macrophages (which typically cannot be achieved using FACS markers alone), we measured protein (ELISA) levels of TNF-α, IL-10, and IL-6 in the culture supernatant as a confirmation of M2b functionality (**Fig. 1H-J**). As expected, M2b(LPS+ICs) macrophages produced high levels of TNF-α, IL-10, and IL-6, compared with the M-CSF control (respectively, *P* = 0.002, CI = 887.0-6,191.0; *P* = 5 × 10^−4^, CI = 1,543.9-4,956.6; *P* = 0.019, CI = 514-10,052). TNF-α concentration was also increased in M1(IFN+LPS), whereas it was decreased for M2a(IL4+IL10) macrophages compared with their controls (**Fig. 1H**; *P* = 0.008, CI = 567.3-5,812.6 and *P* = 0.007, CI = 3.1-31.2). In addition, the IL-10 concentration was increased in M2a(IL4+IL10) macrophages (**Fig. 1I**; *P* = 0.041, CI = 54.7-2,031.0). The IL-6 concentration was also higher in M1(IFN+LPS) macrophages compared with GM-CSF control (**Fig. 1J**; *P* = 0.047, CI = 10.1-2,461.5).

### Effects of Inflammatory Cytokines on FR-β Expression

**Figure 2.**
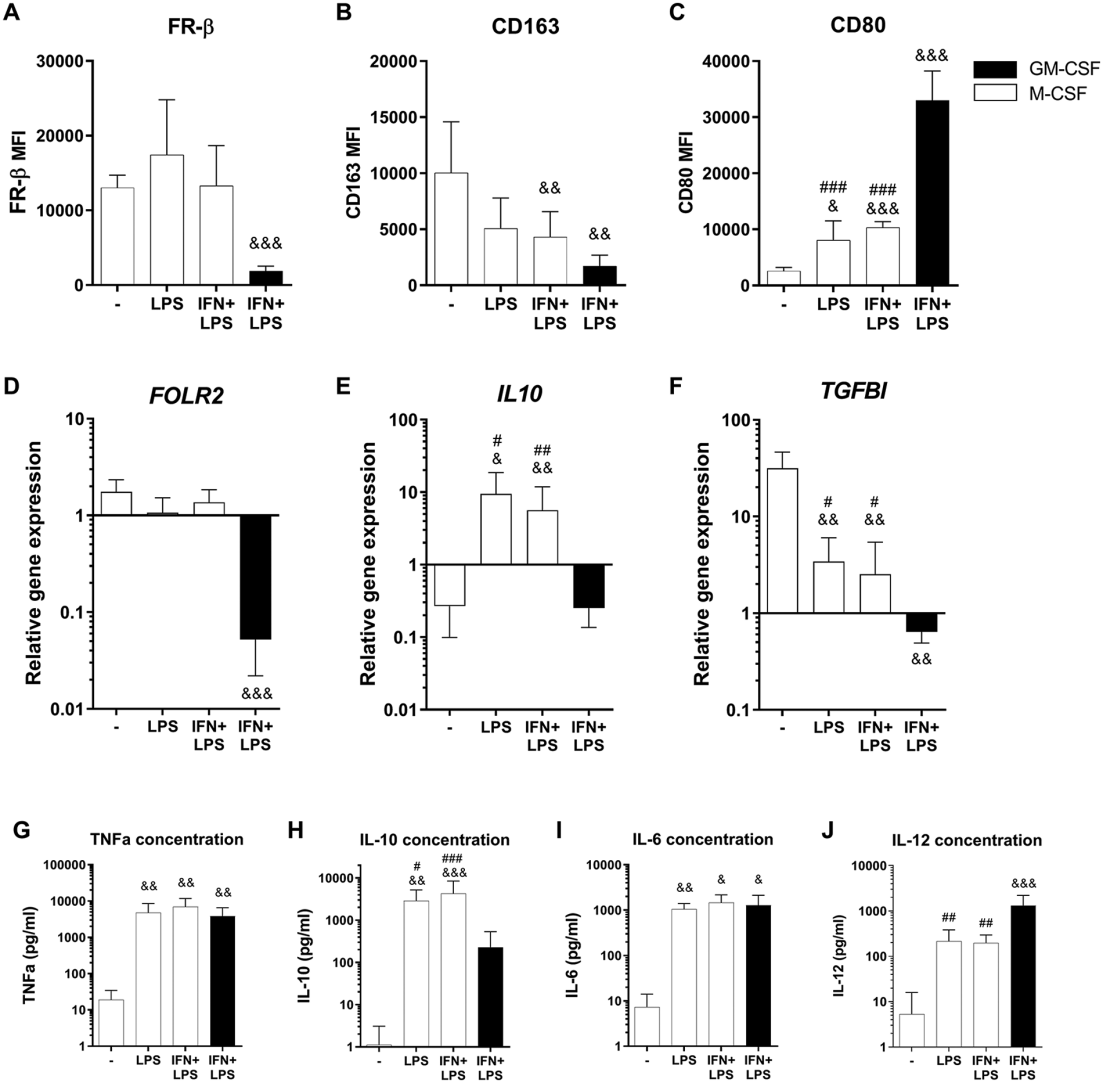
Effects of inflammatory stimulation on FR-β surface expression in M-CSF macrophages. Experimental conditions consist of GM-CSF (black bars) or M-CSF (white bars) generated macrophages, stimulated for 24 hours with corresponding cytokine(s). (**A-C**) Mean fluorescence intensity (MFI) of FR-β, CD163, and CD80 as measured by flow cytometry. (**D-F**) Relative gene expression of FOLR2, IL10, and TGFBI, calculated using delta Ct method and housekeeping genes TBP and YWHAZ. (**H-J**) Cytokine concentration of TNF-α, IL-10, IL-6, and IL-12 in culture supernatants measured by ELISA. Graphs represent mean ± SD. ^&^*P* < 0.05, ^&&^*P* < 0.01, ^&&&^*P* < 0.001 compared with M-CSF control; ^#^*P* < 0.05, ^##^*P* < 0.01, ^###^*P* < 0.001 compared with M1(IFN+LPS), as determined by generalized linear mixed models with Bonferroni correction. FR-β = folate receptor beta; M-CSF = macrophage colony-stimulating factor; GM-CSF = granulocyte-macrophage colony-stimulating factor; TGFBI = transforming growth factor beta induced; TNF = tumor necrosis factor; IL = interleukin; IFN = interferon; LPS = lipopolysaccharide; ELISA = enzyme-linked immunosorbent assay.

It is known that *in vivo* macrophages are more plastic than the typically *in vitro* used subtypes and that they can transform into different subtypes depending on the environmental cues.^9^ Therefore, we set out to investigate what happens to the FR-β expression in M-CSF-generated macrophages, if these macrophages are exposed to an inflammatory environment such as LPS and IFN-γ, instead of further differentiating them using IL4+IL10, LPS+ICs, or TGF-β. We observed that when M-CSF-cultured macrophages are exposed to either LPS or IFN+LPS, the FR-β surface expression remained high, comparable with the M-CSF control macrophages (**Fig. 2A**). This was in contrast to CD163 surface expression, which decreased compared with the M-CSF control, nonsignificantly after exposure to LPS (*P* = 0.37, CI = −957-8,213), but significantly after exposure to IFN+LPS (**Fig. 2B**; *P* = 0.004, CI = 1,059-10,222). In addition, CD80 expression was increased in M-CSF-generated macrophages after LPS and IFN+LPS stimulation (**Fig. 2C**; *P* = 0.01, CI = 745-10,629 and *P* = 2 × 10^−4^, CI = 2,892-12,942, respectively). Altogether, these findings suggested that in M-CSF-generated macrophages both CD163 and CD80 expression changed to a more M1-like phenotype, as would be expected upon stimulation with LPS and IFN-γ, whereas FR-β surface expression remained high.

A similar tendency was observed at the gene expression level in the M-CSF-cultured LPS and LPS+IFN groups, where *FOLR2* expression remained at the same high level as the M-CSF control group (**Fig. 2D**). *TGFBI* expression decreased after LPS and IFN-γ stimulation (*P* < 0.01 for both groups) but was still higher compared with GM-CSF-generated macrophages (**Fig. 2F**; *P* < 0.05). *IL10* gene expression and IL-10 cytokine concentration were elevated in the M-CSF-generated LPS and IFN+LPS groups, compared with the M-CSF control group (*P* < 0.05 and *P* < 0.01, respectively), but also compared higher relative to the M1(IFN+LPS) macrophages (**Fig. 2E** and **H**; *P* < 0.05 for both). The levels of *IL10* gene expression and IL-10 cytokine concentration were similar to what was observed previously for the M2b(LPS+ICs) subtype (**Fig. 1F** and **I**), pointing toward an LPS-induced IL-10 release in all these M-CSF-generated macrophages. TNF-a and IL-6 concentrations were increased in the media of the M-CSF-generated LPS and IFN+LPS groups compared with the M-CSF control and reached comparable levels to those detected for GM-CSF-generated macrophages cultured under the same conditions (**Fig. 2G** and **I**). Only the IL-12 level in the M-CSF-cultured LPS and LPS+IFN groups was still clearly lower than M1(IFN+LPS) macrophages (**Fig. 2J**; *P* < 0.01 for both groups). Overall, of all measured markers only FR-β surface expression and *FOLR2* gene expression did not change in M-CSF-generated macrophages after addition of LPS and IFN-γ. In fact, these M-CSF-generated macrophages, when subjected to treatment with LPS and IFN-γ, obtained an M1-like phenotype, with the exception of both IL-10 and FR-β at gene and protein level.

### Effects of Corticosteroids on Macrophage Polarization and FR-β Expression

To study the effects of an anti-inflammatory environment on plasticity and FR-β expression in M-CSF- and GM-CSF-generated macrophages, the corticosteroid TA was used. TA was supplemented at day 7, for 24 hours, similar to the stimulations in previous culture experiments with IFN and LPS, but also at earlier time points (day 0 and day 3) during the macrophage generation, to examine the effects of early stimulation. Surface marker and gene expression were always analyzed on day 8.

**Figure 3.**
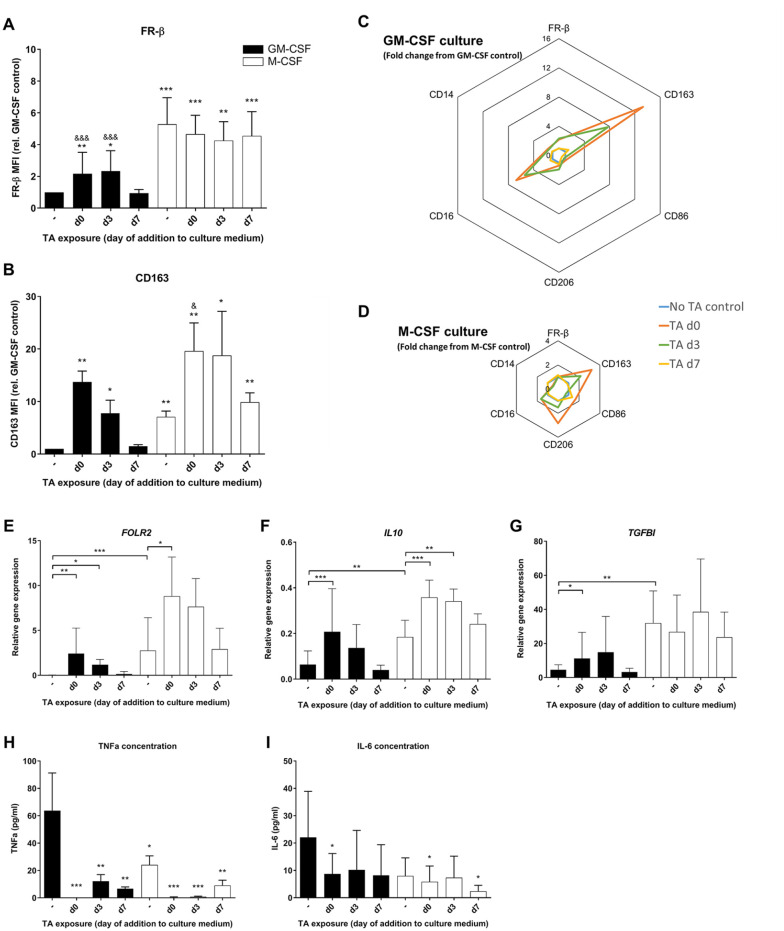
Effects of corticosteroid triamcinolone acetonide (TA) exposure on macrophage FR-β surface expression. Experimental conditions consist of GM-CSF (black bars) or M-CSF (white bars) generated macrophages, with TA added at different time points during culture. (**A, B**) Mean fluorescence intensity (MFI) of FR-β and CD163 relative to the GM-CSF control, measured by flow cytometry. (**C, D**) Fold change in surface marker expression measured by flow cytometry of TA day 0, day 3, and day 7 relative to no TA control, shown separately for GM-CSF- and M-CSF-cultured macrophages. (**E-G**) Relative gene expression of FOLR2, IL10, and TGFBI, calculated using delta Ct method and housekeeping genes TBP and YWHAZ. (**H-J**) Cytokine concentration of TNF-α, IL-10, and IL-6 in culture supernatants measured by ELISA. Graphs represent mean ± SD. *P < 0.05, **P < 0.01, ***P < 0.001 compared with GM-CSF control; ^&^*P* < 0.05, ^&&^*P* < 0.01, ^&&&^*P* < 0.001 compared with M-CSF control, as determined by generalized linear mixed models with Bonferroni correction. FR-β = folate receptor beta; GM-CSF = granulocyte-macrophage colony-stimulating factor; M-CSF = macrophage colony-stimulating factor; IL = interleukin; TGFBI = transforming growth factor beta induced; TNF = tumor necrosis factor; ELISA = enzyme-linked immunosorbent assay.

TA supplementation at day 7 to GM-CSF-generated macrophages did not affect FR-β expression. However, TA supplementation on day 0 or day 3 resulted in almost a 2-fold increase in FR-β expression on day 8 in GM-CSF-generated macrophages (**Fig. 3A**; day 0: *P* = 0.07, CI = 0.2-1.9 and day 3: *P* = 0.043, CI = 0.02-2.1). In accordance with our previous observations, M-CSF-generated macrophages had high FR-β surface expression, and this did not increase further upon supplementation of TA. Further analysis confirmed that TA addition on day 0 or day 3 increased CD163 surface expression in GM-CSF-generated macrophages (**Fig. 3B**; *P* = 0.002, CI = 3.2-20.0 and *P* = 0.018, CI = 0.6-10.8, respectively). For M-CSF-generated macrophages, an increase in CD163 surface expression was observed when TA was added at day 0 (**Fig. 3B**; *P* = 0.04, CI = 3.6-15.9). The fold changes in the surface marker profiles relative to the GM-CSF control without TA (**Fig. 3C**) and relative to M-CSF control without TA (**Fig. 3D**) indicate that for most surface markers the change in expression was largest when TA was added on day 0 and somewhat limited when TA was added on day 3, whereas it remained unaltered when TA was added on day 7. This suggests that there is more plasticity in response to the anti-inflammatory stimulus in early maturation stages at day 0 and day 3 of culture.

The *FOLR2* gene expression in GM-CSF-generated macrophages showed the same increase as FR-β expression upon addition of TA on day 0 or day 3 (**Fig. 3E**; *P* < 0.01 and *P* < 0.05, respectively). In contrast to what was observed for FR-β surface marker in M-CSF-generated macrophages, TA addition at day 0 resulted in an increase in *FOLR2* gene expression compared with the M-CSF control (**Fig. 3E**; *P* = 0.04, CI = 0.3-11.8). TA treatment on day 0 increased gene expression of *IL10* and *TGFBI* in GM-CSF-generated macrophages (**Fig. 3F** and **G**; *P* < 0.001, CI = 0.07-0.23 and *P* = 0.04, CI = 0.4-13, respectively). Furthermore, TA addition decreased TNF-α production of all macrophages (*P* < 0.01); the effect was most prominent when TA was added at day 0 (**Fig. 3H**; *P* < 0.001).

## Discussion

In this study, we show that FR-β expression is strongly induced by M-CSF in monocyte-derived anti-inflammatory M2-like macrophages *in vitro.* In addition, we show that FR-β is induced by TA treatment during macrophage generation, resulting in a CD163^+^ FR-β^+^ (M2-like) anti-inflammatory macrophage phenotype, even in an M1 priming environment with GM-CSF and no M-CSF.

In agreement with our findings, FR-β expression has previously been shown to be higher in anti-inflammatory macrophages and is also high in tumor-associated macrophages (TAM) that have a similar M2-like phenotype.^31,35^ We observed that FR-β expression was lower in IC-induced M2b-like macrophages, as was their expression of CD163 and CD16. In agreement with previous findings,^36-38^ ICs induced production of TNF-α, IL-6, and IL-10 in these macrophages. To the best of our knowledge, this study is the first to investigate FR-β expression in M2b macrophages stimulated with LPS and ICs.

**Figure 4.**
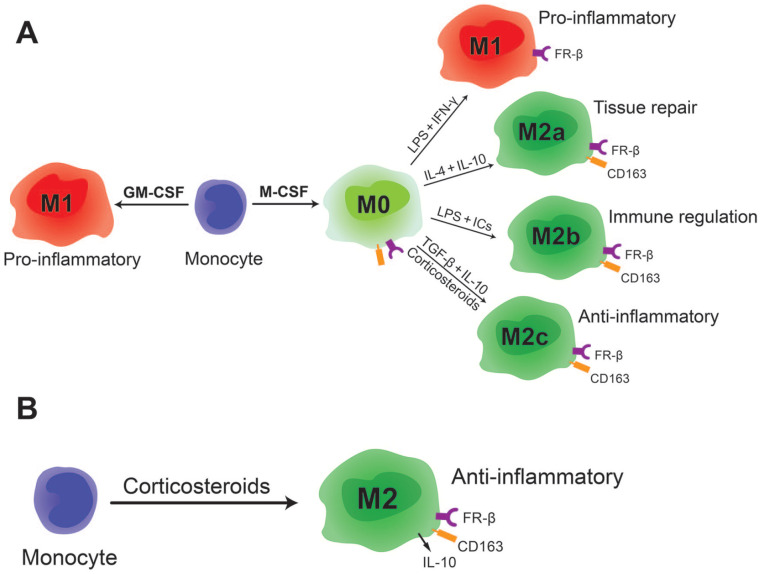
Schematic representation of macrophage phenotypes and their FR-β expression. (**A**) Macrophage subtypes that can be generated *in vitro* by stimulating monocytes with GM-CSF or M-CSF. All M-CSF-generated macrophages are FR-β positive. (**B**) Stimulation of monocytes with corticosteroids generates FR-β-positive anti-inflammatory macrophages. FR-β = folate receptor beta; GM-CSF = granulocyte-macrophage colony-stimulating factor; M-CSF = macrophage colony-stimulating factor.

Macrophages that are generated by M-CSF are known to be plastic and a pro-inflammatory phenotype can be induced in these macrophages by further stimulation with LPS and IFN-γ. For this reason they are also often referred to as M0 (illustrated in **Fig. 4**). Remarkably, we observed that FR-β expression remained high when M-CSF-generated macrophages were switched to a pro-inflammatory M1-like phenotype, unlike the other M2 markers such as CD163. The fact that FR-β remains highly expressed in these M-CSF-generated pro-inflammatory macrophages indicates that FR-β expression is not limited to an anti-inflammatory phenotype. This may be more in line with the complex *in vivo* situation in inflammatory joint diseases, where a monocyte can be exposed to M-CSF during maturation but may also encounter an inflammatory environment in the joint, skewing the macrophage to a pro-inflammatory phenotype that (being exposed to M-CSF before) still remains a high expression of FR-β^+^.

Medical treatment in inflammatory joint conditions may have confounding effects on FR-β expression; therefore, this study investigated the effects of corticosteroids on macrophage plasticity and FR-β expression. We confirmed previous findings that corticosteroids induce an anti-inflammatory phenotype in macrophages by CD163 surface expression, and *IL10* and *TGFBI* gene expression.^11,12,39^ We show that next to these anti-inflammatory markers, TA induces cell surface FR-β expression in GM-CSF-generated macrophages and FR-β gene expression in both M-CSF- and GM-CSF-generated macrophages. In GM-CSF-generated macrophages, the effect of TA mainly occurs in the early stages of maturation, when added on, or before, day 3 of the culture. In this study, we always measured FR-β expression at the end of the culture period on day 8, when the macrophages have fully matured. Interestingly, Puig-Kröger *et al.* found that in culture, upon addition of M-CSF, FR-β became detectable by day 3 and increased further until day 7 of culture.^31^ However, in this context it must also be noted that FR-β expression is shown in some human monocyte populations.^40^ In addition, Xia *et al.* showed inhibition of folate uptake by macrophages when stimulated on day 5 with IFN-γ or LPS.^15^ In the current study, where we added IFN-γ and LPS at day 7 in M-CSF-generated macrophages, we no longer observed any effect on FR-β expression. Together with our finding that TA induces FR-β expression in GM-CSF-generated macrophages when added on, or before, day 3 of culture, this seems to indicate that the FR-β expression in macrophages can only be influenced *in vitro* until day 5 of culture. This is in contrast to other M1/M2-surface markers, such as CD80 and CD163, which can still be modulated after macrophage maturation at 7 days.

FR-β is under evaluation as a clinical marker and target in OA and RA, but ambiguous findings regarding the phenotype of FR-β^+^ macrophages hamper clinical implementation. To date, the phenotype and inherent functionality of FR-β-expressing macrophages *in vivo* is identified as showing both pro-inflammatory and anti-inflammatory characteristics. Our results confirm that these phenomena can be replicated *in vitro* and that FR-β as a marker can represent both pro- and anti-inflammatory phenotypes. FR-β is strongly induced by M-CSF in anti-inflammatory (M2) macrophages. At the same time, FR-β can also be expressed on pro-inflammatory macrophages with an M2 (M-CSF) history, as we observed that M-CSF-generated macrophages maintain their FR-β expression levels after stimulation with LPS and IFN-γ. This could explain why pro-inflammatory FR-β^+^ macrophages are observed in the synovium of OA and RA patients and why FR-β^+^ macrophages are associated with disease symptoms such as pain, joint space narrowing, and the presence of osteophytes.^20,25,41,42^ Likewise, in animal models, FR-β^+^ macrophages accumulate at the site of inflammation, relate to OA and RA disease severity, and can successfully be used as a target for therapy.^15,19,43-46^ However, when TA treatment is administered in a rat OA model, osteophyte formation is prevented, but at the same time FR-β expression is extremely elevated.^12^ This suggests that FR-β expression in the latter model does not reflect disease severity, but rather a corticosteroid induced shift toward FR-β^+^ anti-inflammatory macrophages. This is in line with our observation that TA induces FR-β^+^ GM-CSF-generated macrophages, despite the M1 priming environment with GM-CSF. Apparently, macrophages with an M1 history can become FR-β positive and anti-inflammatory M2-like upon stimulation with corticosteroids. The latter finding could provide new opportunities to monitor the macrophage response to corticosteroid treatment in patients using FR-β-based imaging techniques. In addition, these findings reveal corticosteroid use as an important exclusion criterion for experimental treatment of OA and RA with FR-β-based therapeutic approaches, as targeting of FR-β^+^ anti-inflammatory (M2) macrophages could be detrimental. As long as corticosteroid use is taken into account, FR-β will remain an exciting potential target in clinical applications. On one hand, as shown in many previous studies to monitor disease status and as a target for treatment or, alternatively, to monitor treatment response to corticosteroid therapy.

In conclusion, we show that in an environment without corticosteroids, FR-β is induced by M-CSF and highly expressed by M2-like anti-inflammatory macrophages. In these macrophages, FR-β expression remains high after stimulation with IFN-γ and LPS, resulting in an FR-β^+^ (M1-like) pro-inflammatory macrophage population. When TA is added during macrophage generation, FR-β^+^ (M2-like) anti-inflammatory macrophages will form, despite the absence of M-CSF and the presence of GM-CSF, which would normally result in an M1 priming environment. This effect of corticosteroids on FR-β expression and previous observations made *in vivo*^12^ indicates that the purpose of FR-β-based imaging can be expanded to monitor patient response to corticosteroid therapy. In OA and RA patients who do not receive corticosteroids, FR-β can remain a potential marker for disease severity and treatment target. This is an important observation in view of further developments in FR-β-based diagnostic and therapeutic approaches in inflammatory joint diseases and warrants further investigation.

## Supplemental Material

sj-docx-1-car-10.1177_19476035221081469 – Supplemental material for Folate Receptor Expression by Human Monocyte–Derived Macrophage Subtypes and Effects of Corticosteroids Click here for additional data file. Supplemental material, sj-docx-1-car-10.1177_19476035221081469 for Folate Receptor Expression by Human Monocyte–Derived Macrophage Subtypes and Effects of Corticosteroids by Kelly Warmink, Michiel Siebelt, Philip S. Low, Frank M. Riemers, Bingbing Wang, Saskia G. M. Plomp, Marianna A. Tryfonidou, P. René van Weeren, Harrie Weinans and Nicoline M. Korthagen in CARTILAGE

sj-docx-2-car-10.1177_19476035221081469 – Supplemental material for Folate Receptor Expression by Human Monocyte–Derived Macrophage Subtypes and Effects of Corticosteroids Click here for additional data file. Supplemental material, sj-docx-2-car-10.1177_19476035221081469 for Folate Receptor Expression by Human Monocyte–Derived Macrophage Subtypes and Effects of Corticosteroids by Kelly Warmink, Michiel Siebelt, Philip S. Low, Frank M. Riemers, Bingbing Wang, Saskia G. M. Plomp, Marianna A. Tryfonidou, P. René van Weeren, Harrie Weinans and Nicoline M. Korthagen in CARTILAGE
