# Supplementary material for: Folate Receptor Expression by Human Monocyte–Derived Macrophage Subtypes and Effects of Corticosteroids
Source: Cartilage. 2022 Mar 7;13(1):19476035221081469. doi: 10.1177/19476035221081469 (PMC9137314; doi:10.1177/19476035221081469)
Supplement: sj-docx-1-car-10.1177_19476035221081469 – Supplemental material for Folate Receptor Expression by Human Monocyte–Derived Macrophage Subtypes and Effects of Corticosteroids [file sj-docx-1-car-10.1177_19476035221081469.docx]

| **Supplementary table 1.**  Flow cytometry antibodies | | | | |
| --- | --- | --- | --- | --- |
| **Target** | **Clone** | **Label** | **Producer** | **Dilution** |
| FR-β | M909 | Biotin | Feng et al. [29] | 1:200 |
| CD68 | EBM11 | FITC | DAKO | 1:50 |
| CD86 | IT2.2 | PE | BD Biosciences | 1:20 |
| CD80 | L307.4 | PE | BD Biosciences | 1:20 |
| CD163 | GHI/61 | PerCP-Cy5.5 | Biolegend | 1:100 |
| CD14 | RMO52 | APC-AF750 | Beckman Coulter | 1:200 |
| CD206 | 3.29B1.10 | PC7 | Beckman Coulter | 1:100 |
| CD16 | 3G8 | APC | Life technologies | 1:100 |
