# Supplementary material for: Folate Receptor Expression by Human Monocyte–Derived Macrophage Subtypes and Effects of Corticosteroids
Source: Cartilage. 2022 Mar 7;13(1):19476035221081469. doi: 10.1177/19476035221081469 (PMC9137314; doi:10.1177/19476035221081469)
Supplement: sj-docx-2-car-10.1177_19476035221081469 – Supplemental material for Folate Receptor Expression by Human Monocyte–Derived Macrophage Subtypes and Effects of Corticosteroids [file sj-docx-2-car-10.1177_19476035221081469.docx]

| **Supplementary Table 2.** PCR primer sequences | | | |
| --- | --- | --- | --- |
| **Gene** | **Primers** | **Annealing**  **temp. (°C)** | **Amplicon size (bp)** |
| IL10 | F: 5’- GACTTTAAGGGTTACCTGGGTTG – 3’  R: 5’-TCACATGCGCCTTGATGTCTG– 3’ | 67 | 112 |
| FOLR2 | F: 5’- CAGCAACGGAGGTTCAGC– 3’  R: 5’-CATGGTGGCTACACAGACCA– 3’ | 67 | 95 |
| TGFBI | F: 5’- ATGACCCTCACCTCTATGTAC– 3’  R: 5’-CACAGTTCACAGTTACAATCCCA– 3’ | 67 | 79 |
| TBP | F: 5’- TGCACAGGAGCCAAGAGTGAA– 3’  R: 5’-CACATCACAGCTCCCCACCA– 3’ | 63.5 | 132 |
| YWHAZ | F: 5’- ACTTTTGGTACATTGTGGCTTCAA– 3’  R: 5’-CCGCCAGGACAAACCAGTAT– 3’ | 64 | 94 |
